# Abiotic stressors in poultry production: A comprehensive review

**DOI:** 10.1111/jpn.14032

**Published:** 2024-08-12

**Authors:** Chris Major Ncho, Janine I. Berdos, Vaishali Gupta, Attaur Rahman, Kefala Taye Mekonnen, Allah Bakhsh

**Affiliations:** ^1^ Department of Environmental Systems Science Institute of Agricultural Sciences, ETH Zürich Zürich Switzerland; ^2^ Department of Animal Science College of Agriculture and Forestry, Tarlac Agricultural University Malacampa Tarlac Philippines; ^3^ Division of Applied Life Sciences (BK21 Four Program) Gyeongsang National University Jinju‐si Republic of Korea; ^4^ Department of Medicine and Therapeutics Faculty of Medicine, The Chinese University of Hong Kong Hong Kong China; ^5^ Department of Animal Science College of Agriculture and Environmental Science, Arsi University Asella Oromia Ethiopia; ^6^ Atta‐ur‐Rahman School of Applied Biosciences (ASAB) National University of Sciences and Technology (NUST) Islamabad Pakistan

**Keywords:** chicken, cold stress, duck, heat stress, light, noise, vibration, welfare

## Abstract

In modern animal husbandry, stress can be viewed as an automatic response triggered by exposure to adverse environmental conditions. This response can range from mild discomfort to severe consequences, including mortality. The poultry industry, which significantly contributes to human nutrition, is not exempt from this issue. Although genetic selection has been employed for several decades to enhance production output, it has also resulted in poor stress resilience. Stress is manifested through a series of physiological reactions, such as the identification of the stressful stimulus, activation of the sympathetic nervous system and the adrenal medulla, and subsequent hormonal cascades. While brief periods of stress can be tolerated, prolonged exposure can have more severe consequences. For instance, extreme fluctuations in environmental temperature can lead to the accumulation of reactive oxygen species, impairment of reproductive performance, and reduced immunity. In addition, excessive noise in poultry slaughterhouses has been linked to altered bird behaviour and decreased production efficiency. Mechanical vibrations have also been shown to negatively impact the meat quality of broilers during transport as well as the egg quality and hatchability in hatcheries. Lastly, egg production is heavily influenced by light intensity and regimens, and inadequate light management can result in deficiencies, including visual anomalies, skeletal deformities, and circulatory problems. Although there is a growing body of evidence demonstrating the impact of environmental stressors on poultry physiology, there is a disproportionate representation of stressors in research. Recent studies have been focused on chronic heat stress, reflecting the current interest of the scientific community in climate change. Therefore, this review aims to highlight the major abiotic stressors in poultry production and elucidate their underlying mechanisms, addressing the need for a more comprehensive understanding of stress in diverse environmental contexts.

## 1 INTRODUCTION

Stress in animals can be defined as a physiological and psychological response to external and internal factors that disrupt an animal's homoeostasis or normal physiological balance (Clark et al., 1997). These factors may include habitat challenges, temperature changes, food availability, social interactions, and exposure to predators or other threats (Karaer et al., 2023). In response to stress, animals typically experience a series of physiological changes, including the release of stress hormones such as cortisol in mammals or corticosterone in birds. These hormones can have far‐reaching effects on the animal's body and are aimed at helping the animal cope with immediate challenges. However, chronic or excessive stress can have detrimental effects on an animal's health and well‐being (Fourie et al., 2016). Common signs of stress in animals include altered behaviour, changes in appetite, weight loss, reduced reproductive success, compromised immune function, and increased susceptibility to disease (Chen et al., 2015; Morey et al., 2015; Scott‐Solomon et al., 2021).

In the context of poultry farming, a multitude of factors contribute to creating a complex web of stressors. For example, environmental stressors such as temperature fluctuations, humidity levels, air quality, and lighting conditions can significantly impact the health, growth, and productivity of poultry (Nawab et al., 2018). For instance, Iraqi et al. (2024) stated that thermal stress negatively impacts poultry welfare and productivity. The authors further described that thermal manipulation during egg incubation and exposing eggs to higher temperatures (39.5°C with 60% RH) from Days 12 to 18 improved embryonic development, hatching characteristics, and chick quality. Posthatch, these chicks exhibited better thermos tolerance and higher body weight. The study recommends thermal manipulation during Days 12 to 18 of incubation for its positive effects on both pre‐and posthatch performance, enhancing productivity and welfare in poultry. In a similar study, Soliman et al. (2023) using LED lighting instead of incandescent bulbs for broiler breeder hens significantly improved productivity, including body weight, feed conversion, and egg production. These benefits persisted into the production period, making LED lighting a highly effective option for enhancing poultry productivity and welfare. Extreme variations in temperature can lead to heat or cold stress, negatively affecting birds' comfort and performance. Poor air quality can result in respiratory issues among the birds, while inadequate lighting can disrupt their natural biological rhythms (Lara & Rostagno, 2013). Similarly, managerial stressors are closely linked to the management practices employed on poultry farms and encompass various aspects such as housing design, handling procedures, and nutritional strategies. Overcrowded or poorly ventilated housing conditions can create a stressful environment and promote disease outbreaks. Improper handling during transportation or routine procedures can also have adverse effects on bird welfare (Averós et al., 2020). Nutrition is a vital consideration, as imbalanced diets can lead to poor growth and reduced production efficiency. Effectively managing these stressors is crucial for ensuring the overall well‐being of poultry and optimizing production outcomes (Lashari et al., 2023).

To make informed decisions and develop effective strategies for managing and mitigating potential stressors, it is essential to understand their complexity. Thus, the objective of this review was to comprehensively examine abiotic stressors that impact poultry farming. While pathogens are numerous and complex, abiotic stressors are relatively well‐known in the industry. Hence, in the following sections, a comprehensive overview of the stress physiology in animals will be presented, followed by a discussion of the major abiotic stressors affecting the modern poultry industry.

## 2 COORDINATION OF STRESS RESPONSE IN ANIMALS

**Figure 1:**
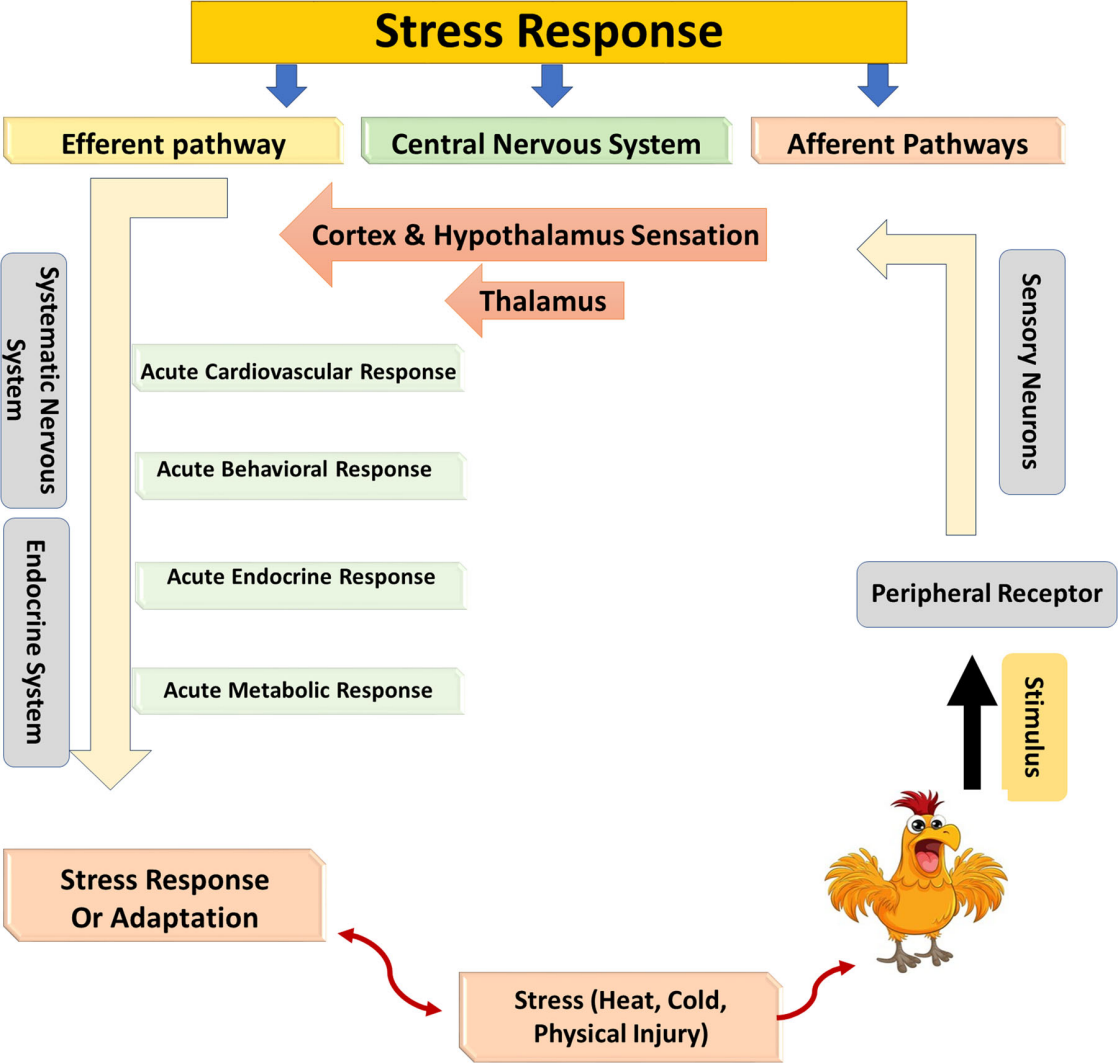
Summary of the mechanism of the stress response in animals (poultry as a model). The response of animals to stressors involves the recognition and communication of perceived stimuli, which activates neurophysiological processes that initiate a physiological reaction. This organized reaction includes the involvement of the central nervous system, endocrine system, and immune system, and has an impact on the animal's behaviour in dealing with the experienced challenge. [Color figure can be viewed at wileyonlinelibrary.com]

The integration of stress within animals is a complex and highly coordinated process that involves various physiological and neural systems working together to respond to perceived threats or challenges. This stress response encompasses the body's reaction to both internal and external stressors, leading to a series of interconnected events within an animal's body (Karaer et al., 2023) The reaction to stressors begins with the detection and signalling of a perceived threat within an animal's biological mechanisms. Subsequently, this triggers the activation of neurophysiological processes aimed at initiating a biological response to resist and mitigate potential harm (Calefi et al., 2017) Notably, the various sensory detectors not only receive information about the stressor but also convert this data into neural signals. These signals are then transmitted to both cognitive and noncognitive centres of the nervous system, facilitating the generation of a synchronized reaction to the encountered challenge. This coordinated response involves the interplay between the central nervous system (CNS), the endocrine system, and the immune system, which collectively react to stressful stimuli and influence an animal's behaviour, as depicted (Figure 1). The presence of common hormones, neurotransmitters, and receptors across these three systems underscores the existence of communication and interaction among them (Von Borell, 2001).

The initial perception of a stressor elicits a complex response in animals, relying on their sensory systems to detect environmental changes or potential threats. This sensory information is then relayed to specific regions of the brain, such as the amygdala and prefrontal cortex, which evaluate the significance and potential danger of the stressor. These brain regions play a pivotal role in determining whether a stress response is warranted (Mariotti, 2015). Once the brain identifies a stressor as significant, it triggers the activation of the hypothalamus‐pituitary‐adrenal (HPA) axis, a fundamental regulatory system in the body. The hypothalamus, a small but crucial brain structure, releases corticotropin‐releasing hormone (CRH), which is carried through the hypophyseal portal blood system to reach the anterior pituitary. Within the anterior pituitary, it promotes the synthesis and release of hormones, including adrenocorticotropic hormone (ACTH), β‐endorphin, β‐lipotropin, and α‐melanocyte‐stimulating hormone/α‐melanotropin (Dovolou et al., 2023; Karaer et al., 2023). Elevating glucocorticoid levels in the bloodstream can potentially restrain the secretion of ACTH from the pituitary gland, representing a feedback control mechanism. Nevertheless, the release of ACTH remains contingent upon the stressor's intensity and is influenced by a finely tuned, sensitivity‐based feedback system. Mild stressors may consequently experience gradual inhibition through glucocorticoid feedback, whereas more severe stressors are less likely to be suppressed (Biddie & Hager, 2009; Cockrem, 2013).

The release of stress hormones, such as corticosterone, has profound and widespread effects throughout the body. These physiological changes are aimed at preparing the animal for an immediate response to the perceived threat. For instance, increased glucose levels in the bloodstream provide a rapid source of energy for the “fight or flight” response (Chen et al., 2015). Additionally, heart rate and blood pressure rise, ensuring sufficient oxygen delivery to critical tissues. The respiratory system responds with dilated bronchioles, facilitating enhanced oxygen intake. However, the digestive system experiences temporary alterations as digestion and nutrient absorption are halted, redirecting resources elsewhere (Karaer et al., 2023; Von Borell, 2001). Stress can also temporarily suppress the immune system's response, rendering animals more susceptible to infections. Both physical and psychological stressors have been shown to suppress the activity of T‐ and B‐lymphocytes, as well as natural killer cells. Moreover, they can reduce the production of certain cytokines, including interleukin‐2 (IL‐2) and interferon‐γ (IFN‐γ). Cytokines, such as IL‐1, IL‐6, tumour necrosis factor‐α (TNF‐α), and IFN‐γ, play crucial roles as mediators in the immune and pathological responses triggered by both stress and infection (Sonnenfeld et al., 1992).

Stress can also lead to heightened emotional responses and the formation of emotional memories associated with the stressful event. However, the hippocampus, a vital brain region, plays a balancing role by providing negative feedback to the HPA axis, helping to terminate the stress response once the perceived threat subsides (Rich & Romero, 2005; Sheng et al., 2021). Chronic or recurring exposure to stressors can have long‐term effects on an animal's neurobiology, resulting in structural changes in the brain, particularly within regions like the amygdala and hippocampus (Terio et al., 2004; Wiechers et al., 2021).

As demonstrated, stress appears to trigger a series of reactions within organisms. However, depending on the source and intensity of the stress, some reactions may be more pronounced than others, with inhibition or stimulation of specific pathways. According to current literature, factors such as temperature, noise, vibration, and light intensity are among the most frequently reported abiotic stressors in poultry farming. Therefore, it is essential to investigate how these factors modulate birds' responses, as this can have significant implications for the industry.

## 3 TEMPERATURE‐RELATED STRESSORS

**Figure 2:**
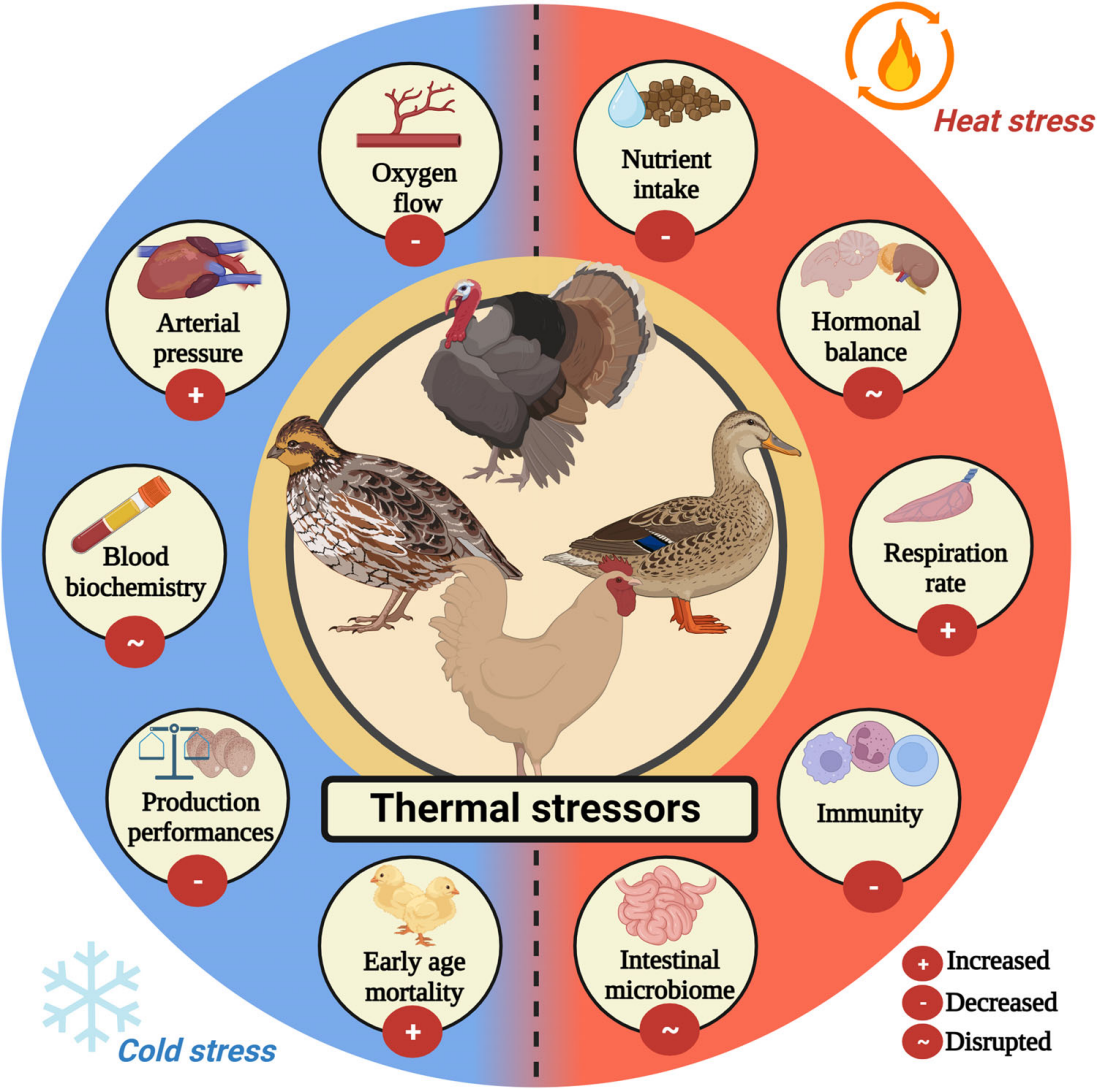
Selected established or suggested effects of thermal stressors on the poultry. Poultry may exhibit behavioural, physiological, and immunological responses in response to both cold and heat stress, which can have negative impacts on their health and well‐being. Ambient temperature fluctuations can significantly impact the metabolism of birds, either by increasing or decreasing their energy expenditure or by disrupting their normal physiological processes. [Color figure can be viewed at wileyonlinelibrary.com]

In the context of poultry, temperature plays a pivotal role throughout their development, from the embryonic stage to maturity (Tzschentke, 2008). The common fowl species have a limited ability to regulate their body temperature, especially when the environmental temperature does not fall within the narrow range of 16–26°C. This characteristic makes poultry, especially modern strains resulting from years of selective breeding, vulnerable to both high and low ambient temperatures (Ncho et al., 2022b). For instance, during their embryonic development, many species used in animal production require temperatures exceeding 36°C (Visschedijk, 1991). Also, deviations from the optimal temperature, such as exposure to low temperatures in the first week after hatching, can lead to severe health issues and, ultimately, mortality (Ncho, Gupta, et al., 2021; Yerpes et al., 2020). In contrast, broilers, for example, exhibit optimal growth between 20°C and 24°C after 27 days of age. Thus, understanding the physiological impact of extreme temperature variations on birds is crucial. Both heat and cold stress have deleterious effects (Figure 2), emphasizing the importance of understanding the complex relationship between temperature fluctuations and avian physiology (Bilal et al., 2021).

### 3.1 Heat stress in poultry

Heat stress arises when an organism's natural cooling mechanisms struggle to effectively dissipate heat, leading to a noticeable rise in the body temperature (Goel, Ncho, et al., 2021; Ncho, Jeong, et al., 2021). Unlike mammals, birds lack the ability to sweat, severely limiting their capacity to cool down in high‐temperature environments (Ncho et al., 2022a; Ncho, Goel, et al., 2021). During episodes of heat stress, there is a complex interplay of behavioural, immunological, and physiological changes, profoundly impacting the health and productivity of avian species (Goel, 2021). These alterations in response to elevated temperatures are multifaceted and include a range of metabolism‐related shifts (Saeed et al., 2019).

#### 3.1.1 Behavioural responses of poultry to high ambient temperatures

One significant behavioural adjustment observed in birds enduring heat stress involves modifications in their locomotor activities (Wang et al., 2018). To minimize heat generation, birds tend to limit their movement, preferring to remain in a stationary position. Noteworthy findings from a study utilizing comprehensive 24‐h camera monitoring revealed that broilers exposed to prolonged high ambient temperatures predominantly adopted a sedentary posture, rising only when necessary, primarily to access water sources (Branco et al., 2021). Furthermore, feed intake is noticeably reduced, mainly due to the heat generated during the consumption process. This reduction in feed intake not only hinders growth but also severely restricts essential nutrient absorption, affecting overall development and productivity (Abdel‐Moneim et al., 2021). Conversely, specific behaviours are intensified during heat stress episodes. For instance, there is an increase in water consumption among heat‐stressed birds (Ahmad et al., 2008). Recent trials have highlighted that broilers subjected to cyclic heat stress conditions elevate their water‐to‐feed ratio by more than 30%, emphasizing the crucial role of hydration in mitigating the effects of heat stress (Bruno et al., 2011). The higher water intake is vital in maintaining optimal body water content, ensuring the facilitation of vital physiological functions despite the challenging thermal conditions (Beker & Teeter, 1994; Borges et al., 2004). Additionally, observations indicate that birds tend to adopt specific postures to enhance cooling (Zulkifli et al., 1999). Spread wings and excessive panting become common behaviours, especially when temperatures exceed the birds' optimal range (Branco et al., 2021; Li et al., 2015). In systems without cages, birds instinctively cover themselves with litter, creating a layer that aids in evaporative cooling. These nuanced behavioural adaptations play a pivotal role in the birds' ability to cope with heat stress, underscoring the specific means by which avian species respond to environmental challenges.

#### 3.1.2 Physiological responses of poultry to high ambient temperatures

The second category of changes associated with heat stress are physiological. Physiological changes in a bird's body can significantly disrupt various bodily functions and processes. One critical aspect of these physiological alterations is the induction of oxidative stress due to heat. Oxidative stress arises from an imbalance between free radicals and the body's antioxidant system (Akbarian et al., 2016; Ncho et al., 2023). Normally, cells produce reactive oxygen species (ROS) during their regular activities, but these ROS are typically eliminated by detoxifying mechanisms within the body (Surai et al., 2019). However, heat stress disrupts this balance in two ways: it either increases the production of ROS or reduces the effectiveness of the body's first line of defence, namely catalase (CAT), glutathione peroxidase (GPx), and superoxide dismutase (SOD) (Surai, 2020). SOD plays a pivotal role by catalyzing the conversion of superoxide radicals into hydrogen peroxide and oxygen (Surai, 2016, 2020). Subsequently, CAT transforms hydrogen peroxide into harmless water and oxygen (Liu et al., 2007), while GPx reduces organic hydroperoxides into corresponding alcohols using glutathione (Surai et al., 2018). When there's an excess of ROS in the body due to prolonged heat exposure, these molecules damage essential cell components such as proteins, lipids, and especially DNA (Bandyopadhyay et al., 1999). The severity of heat‐induced oxidative stress depends on factors such as the intensity and duration of exposure (Habashy et al., 2019; Ncho et al., 2023). While the levels of antioxidant enzymes may initially rise during the onset of heat stress, prolonged exposure inevitably leads to chronically reduced enzyme levels, exacerbating the damaging effects of oxidative stress on the body's cells (Goel, 2021; Goel, Ncho, et al., 2021; Gupta et al., 2024).

Another physiological change associated with heat stress is the disruption of acid‐base balance (Borges et al., 2007). As a result of panting, birds increase their respiration rate and evaporative cooling from their lungs (Toyomizu et al., 2005). This often leads to higher excretion of carbon dioxide via the respiratory route, exceeding the cellular production of carbon dioxide (Collier et al., 1982). Consequently, the standard bicarbonate buffer system in the blood undergoes alterations (Bottje et al., 1989). Specifically, levels of carbonic acids and hydrogen ions decrease, while bicarbonate ion levels remain high (Odom et al., 1986). This phenomenon, known as respiratory alkalosis, profoundly impacts renal function (Teeter et al., 1985). The imbalance in the blood's acid‐base status has been linked to reduced performances in both broilers and layers (El Hadi & Sykes, 1982). While the reduction in performance is a result of multiple factors, it is also greatly influenced by feed intake (Cooper & Washburn, 1998). The physiological explanation for decreased intake during heat stress is attributed to hormonal changes (Mazzoni et al., 2022). The neuronal‐hypothalamic axis plays a crucial role in regulating feed intake and energy homoeostasis (Goel, Ncho, et al., 2021). Various hormones are involved in maintaining feed intake and energy balance through the neuronal‐hypothalamic axis (Richards & Proszkowiec‐Weglarz, 2007). Previous studies have demonstrated that in poultry, Ghrelin and cholecystokinin are the initial hormones involved in appetite regulation (Honda et al., 2017; Sirotkin et al., 2013). mRNA levels and concentrations of grelin and cholecystokinin were significantly modified in the blood and major portions of the gastrointestinal tract of broilers and layers following exposure to heat stress (He et al., 2018; Lei et al., 2013; Mazzoni et al., 2022). These hormonal changes have also been closely linked to the levels of CRH (Liu et al., 2012). Prolonged exposure to stress triggers the activation of the HPA axis, leading to the secretion of CRH from the hypothalamus (Nawab et al., 2018). This initiates a cascade of hormonal responses, wherein the pituitary gland is activated to release ACTH (Ncho et al., 2023). ACTH, in turn, stimulates the production and release of corticosteroids by the adrenal glands (Bureau et al., 2009). Consequently, it explains why corticosteroids, especially corticosterone have been used as biomarkers of heat stress in poultry (Iqbal et al., 1990; Kim & Choi, 2014).

#### 3.1.3 Heat stress impacts on immunity and gut health

The impact of heat stress on the endocrine system results in immunosuppression, a direct consequence of this physiological modification (Bilal et al., 2021; Jahanian & Rasouli, 2015). The immune response is tightly regulated by the CNS through a complex network involving the nervous, endocrine, and immune systems (Dantzer, 2018). In recent years, extensive research has explored the detrimental effects of heat stress on poultry, consistently revealing a decline in immune functions. Specifically, continuous exposure to elevated ambient temperatures has been linked to reduced lymphoid organ indexes, indicating a compromised immune system (Rotiah et al., 2019). Moreover, lower relative liver weights in heat‐stressed laying hens and a drastic decrease in circulating antibodies, particularly IgM and IgA, have been observed in broilers subjected to environmental temperatures above the recommended range (Attia et al., 2016; Awad et al., 2020). Regarding cell‐mediated immune response, researchers have documented reduced phagocytic activity of macrophages, lower T lymphocyte counts, and decreased expression of interleukins in broilers facing heat stress challenges (Hirakawa et al., 2020; Park et al., 2013). It is proposed that the higher levels of corticosteroids and catecholamines observed during heat injury can impair the proliferation of immune cells and antibody production, which may explain the reduced size of lymphoid organs (Goel, 2021).

It has been established that heat stress in poultry can have a significant impact on the systemic immune response, making birds more susceptible to pathogens, particularly through the gastrointestinal tract (Brugaletta et al., 2022). The gastrointestinal tract of poultry is composed of mucosa and a single layer of cells called enterocytes, which are held together by tight junctions (Ricke et al., 2020). The humoral and cellular‐mediated components of the immune system act as the first line of defence against pathogens in the intestine. During heat stress, the decrease in oxygen availability, inflammation, and limited feed intake can impair the integrity of the intestinal barrier, leading to limited production of digestive enzymes, which can cause oxidative stress and damage to the mucosa (Goel, 2021). Previous studies have found that heat stress can cause chronic injuries to the intestinal walls, sloughing of the head of villi, and shedding of epithelial cells (He et al., 2019; Liu et al., 2020; Nanto‐Hara et al., 2020). Additionally, heat stress has been found to reduce the expression of genes such as occludin and zonula occludens, which are barrier‐related molecules responsible for the junctions between enterocytes in the gut (Cheng et al., 2019). As a result, intestinal permeability increases, allowing pathogens such as *Clostridium spp*. and *Campylobacter* to infiltrate the bloodstream (Rostagno, 2020). Recent studies using advanced sequencing techniques have also confirmed that the microbial diversity and composition of the gut are significantly altered during HS, as the relative abundance of harmful bacteria drastically increases. Apart from the proliferation of pathogenic bacteria, HS has also been linked to changes in microbial taxa abundances, ranging from the phylum to the species level. Notably, long‐term exposure to HS has been shown to reduce the abundance of *Bacteroidetes* in the cecal microbiome of broilers (Goel et al., 2023). This reduction is believed to be mediated by the lower *Bacteroidetes* population observed in the small intestine, as there is evidence of their carbohydrate‐active enzyme encoding capabilities (Flint et al., 2012). Additionally, others have reported an increase in Verrucomicrobia abundance following HS, which is associated with the degradation of mucin gene expression in broilers (Goel et al., 2023). Trials assessing the gut microbiota of other poultry species, in addition to chickens, have also corroborated HS‐related microbial shifts. At lower taxonomic levels, HS has been shown to increase the abundance of already largely dominant *Lactobacillus* genera in the small intestine of ducks, highlighting the lower evenness in microbial species linked to HS previously mentioned in chickens (Tian et al., 2020). In quails, similar results were reported, with HS not only decreasing species richness and evenness but also identifying *Verrucomicrobia* as microbial biomarkers (Qin et al., 2023). While current knowledge suggests that HS has a significant impact on gut health, further research is still ongoing.

### 3.2 Cold stress in poultry

Cold stress can occur in poultry as a result of exposure to temperatures below the recommended range for rearing. Unlike heat stress, which is a well‐documented phenomenon, cold stress appears to be less prevalent in current literature. Indeed, due to the global increase in the temperature of our planet, the current research trends are directed towards enhancing the resistance of the birds to this rising temperature. However, it is crucial to acknowledge that cold stress can have detrimental effects on the welfare, growth, and behaviour of poultry. In particular, it is a significant environmental stressor in certain countries. For instance, in China, more than 120 million bird losses were recorded in the winter of the year 2008 (Nguyen et al., 2016).

#### 3.2.1 Physiological and immunological responses to cold stress

Newly hatched chicks are particularly susceptible to cold stress, especially in the days following hatching, as they have a limited ability to regulate their body temperature. Although this deficiency improves significantly 1 week after hatching, due to better insulation and increased metabolic heat production, cold adaptation can only occur to a limited extent (Wekstein & Zolman, 1971). Prior research has established that exposure to low ambient temperature at a young age can have a severe impact on the physiology of chicks. For example, subjecting broilers to a temperature of 4°C for 24 h has been found to induce alterations in certain genes associated with lipid metabolism in the pituitary, revealing that cold stress has the potential to influence lipid metabolism (Chen et al., 2012). Furthermore, exposing chicks to cold stress has been shown to alter body heat production and increase the expression of genes such as avUCP, AMPKα, and PPARα, which are associated with heat production and fatty acid metabolism respectively (Zhang et al., 2014). Other physiological changes have also been reported, including decreased oxygen consumption, body temperature, respiration, and respiratory water losses. Reduced oxygen intake during cold periods can potentially lower the partial pressure of oxygen in the birds' blood (Bilal et al., 2021). Moreover, low air temperatures necessitate increased oxygen requirements, elevated blood flow, and heightened cardiac output, leading to elevated pulmonary arterial pressure and placing a burden on the right ventricle (Blahova et al., 2007). Cold stress further exacerbates the metabolic oxygen demand (Aarif & Mahapatra, 2013).

It is widely recognized that various abiotic stressors can exacerbate the severity of infectious diseases (Sulmon et al., 2015). Consequently, cold stress likely leads to similar outcomes, even though the underlying mechanisms may not have been fully elucidated. It is proposed that cold stress can modify the immune response of birds by simultaneously influencing bioenergetic and endocrinal pathways (Hangalapura, 2006). In fact, in poultry, immunological functions and thermoregulatory functions are believed to be fuelled by the same energy sources. Therefore, during cold exposure, the increased energy demand for thermoregulation may potentially limit the energy available for proper immune system functioning (Demas et al., 1997). This could result in a reduction in immune cell production and hindered immunological reactions. However, the effects of cold stress on specific immune responses appear to be inconsistent. For instance, Hesters et al. (1996) reported a decline in antibody responses in single‐caged hens following exposure to cold stress. However, such reductions were not observed in hens housed in colony cages. On the other hand, Regnier et al. (1980) found minimal effects of acute cold stress on antibody titers to sheep red blood cells in both broiler and layer chickens. Dabbert et al. (1997) did not detect any signs of compromised humoral immunocompetence following experimentally induced cold stress in Northern bobwhite. Conversely, cold exposure had a negative impact on humoral immunocompetence in blue tits (Svensson et al., 1998) and layer chickens (Gross, 1988). Cold stress also led to depressed cellular immunity in cockerels (Regnier & Kelley, 1981), while growing layer chicks showed an enhancing effect of cold stress on cellular immunity (van Loon et al., 2004).

#### 3.2.2 Cold stress effects on production performances

The effects of low ambient temperatures on the production performance of birds are significant and widespread, regardless of whether they are raised for meat or egg production (Hu & Cheng, 2021; Ipek & Sahan, 2006). For instance, prolonged exposure to cold stress for 72 h can result in a drastic reduction in growth and an increased feed conversion ratio (Zhou et al., 2021). Similarly, the negative effects of cold exposure are exacerbated when broilers are subjected to cold stress for several days, indicating that the duration of exposure is a critical factor to consider during cold stress (Balog et al., 2003; Wei et al., 2018). These reductions in growth during cold stress are thought to be linked to the increased energy expenditure required to maintain homoeothermy (Pani & Bal, 2022). Similarly, layers require substantial energy intake and high mineral requirements for egg production, and low temperatures have been found to increase daily feed intake while simultaneously reducing egg production in commercial laying hens, resulting in poor feed efficiency (Alves et al., 2012; Li et al., 2020). Other studies have also reported concomitant weight loss and reduced egg production in Bedouin hens following cold stress exposure (Spinu & Degen, 1993). While extreme temperatures have been shown to impact production performance, the effects of cold stress appear to be characterized by an inverse relationship between feed intake and production output. Therefore, further research is needed to assess the feeding behaviour of birds during low temperatures in order to enrich the currently limited literature on this topic.

## 4 NOISE STRESS IN POULTRY PRODUCTION

Noise is defined as unwanted or disruptive sound which can be continuous or sporadic. Aesthetically, noise is differentiated from music as the former is unintentional and undesirable while the latter is pleasing to hear (Novak, 2015). Noise is a potential source of stress for animals, as evidenced by a range of studies (Agnes et al., 1990; Forsling, 1984; Stephens et al., 1985; Talling et al., 1996). In particular, noise has been shown to elicit a fear response in animals and has been documented as a stressor in pigs (Bond, 1963; Talling et al., 1998), cattle (Arave et al., 1991; Grandin, 1996; Waynert et al., 1999), and poultry (Campo et al., 2005). Several factors play a role in determining the effect of noise on the productivity and behaviour of animals. However, intrinsic animal‐related factors such as the auditory capabilities of different species and breeds, not to mention the age and physiological condition of the animal at the time of exposure also play a vital role in determining the responses elicited. Characteristics of the noise such as intensity (dB), frequency (Hz), duration, and the ability to produce vibration are important to consider while studying the detrimental effect of noise on animals (Algers et al., 1978). Noise possesses the capability to generate vibrations, prompting the authors to undertake a comprehensive study of their adverse effects on poultry. Interestingly, when a majority of farm animals are capable of hearing frequencies higher than human beings, birds are most responsive to sounds at 2.6 dB–2 kHz (Olczak et al., 2023). Studies show that domestic chickens produce sound ranging from 50 to 10 kHz (Tefera, 2012). In an early study, it was found that at a frequency between 1 and 10 kHz, birds were more sensitive to hearing compared to humans (Kreithen, 1980). Also, birds showed well‐developed abilities to process sound signals and were sensitive to low‐frequency sound stimuli. In fact, a chicken embryo starts perceiving endogenous cochlear signals as early as 12th day of incubation. This period has been referred to as the prehearing period of the birds (Ciborowska et al., 2021). Starting from the 16th day of incubation, the cochlea becomes capable of detecting and encoding sound (Jones et al., 2006). Gradually, as the chicks grow their sensitivity to high‐frequency sound increases (Mihina et al., 2012; Rebillard & Rubel, 1981).

In any animal farm, there can be two major originators of sound. One such source of noise could be the diverse machinery and equipment utilized on the farm, while another originates from the animals themselves. Various devices involved in routine farm management, such as feed dispensers, ventilators, and fans, as well as equipment used by human workers, including cart pullers, washers, and the conversations among farm staff, exemplify sources of sound generated within the animal houses. The movement of large animals, often tethered with metallic chains, can generate considerable noise as they move about. Moreover, activities such as chewing, biting, shifting posture, and vocalizing by animals also contribute to the acoustic environment (Mihina et al., 2012). Between 2008 and 2010, studies on poultry farms housing egg‐laying hens, broilers, and pullets revealed that feed suppliers and distributors were the primary sources of noise within the farm environment (Oh et al., 2011). Another study revealed that broiler chickens are exposed to high‐intensity sounds during the abattoir processing (Chloupek et al., 2009). While the effects of noise on poultry have not been fully investigated, studies have shown that it can negatively impact bird behaviour and production performance (Borg, 1981; Maxwell, 1993; McFarlane & Curtis, 1989; McFarlane, Curtis, Shanks, et al., 1989).

### 4.1 Effect of noise on production and behaviour

Poor welfare conditions have been reported in poultry slaughterhouses due to excessive noise (Geverink et al., 1998; Grandin, 1996). According to Campo et al. (2005), noise can negatively impact the production performance and behaviour of birds. A sound intensity of 115 dB interrupted the brooding of hens (Stadelman, 1958b). However, in the same study, the sound intensity of 96 dB did not affect hatchability or the quality of chicks produced. In another experiment (Stadelman, 1958a), researchers observed that chicks exposed to noises of 80–118 dB showed no effect on growth, but those exposed to noises at 100–118 dB experienced crowding. McFarlane, Curtis, Shanks, et al. (1989) reported that noise had no adverse effect on body weight gain, feed intake, or behaviour traits in broilers. However, Chloupek et al. (2009) found that noise exposure at a level of 100 dB decreased the number of attempts to induce tonic immobility (TI) in broilers. One of the earliest studies to investigate the impact of noise on layers was conducted by Hamm (1967), who found that short‐term exposure to aircraft noise did not negatively affect egg production. However, prolonged periods of stress caused by noise resulted in a decrease in egg production. Campo et al. (2005) also discovered that the duration of TI was significantly longer in hens exposed to noise at an intensity of 90 dB for 60 min. On the other hand, Oh et al. (2011) found that noise did not have any effect on the productivity of laying hens, while vibrations greater than 1.0 cm s^−1^ did affect productivity in layers. However, the habituation of turkeys to stimulated and actual flight noise showed no differential effect on growth (Bradley et al., 1989). Another study conducted on Japanese quail by Woolf et al. (1976) found that exposure to auditory stimulation of 80 dB for 2 h during the last 3 days of incubation accelerated the hatching of chicks.

Typically, domestic fowl respond with head‐turning and a brief startle reflex when exposed to sudden loud noises. However, birds exhibit a remarkable ability for swift habituation, quickly returning to their natural activities within minutes after the acoustic stimulus ceases (Gladwin et al., 1988). The fear response elicited in hens was heightened in birds exposed to 90 dB sound caused by trucks, trains, and aircraft for 1 h compared to hens housed in environments with 65 dB sound primarily from vocalization (Campo et al., 2005). In chickens, exposure to 95 dB sound stimuli at 500 Hz elicited a startling response characterized by a latent period, running, total immobility, small jerky movements of the head, and drowsy behaviour (Algers et al., 1978). It is crucial to rear poultry in an environment with minimal occurrence of loud and sudden noises, as such conditions are known to induce hysteria in various strains of chickens (National Research Council (US) Committee for the Update of the Guide for the Care and Use of Laboratory Animals, 2010). In 10 of the 21 studies, flocks of mature hens aged around 35 weeks showed feather‐pecking behaviour when exposed to recorded machinery and vocalization sounds (Bright, 2008). In other poultry species such as wild turkeys, Lynch and Speake (1978) reported no abnormal behaviour when exposed to sonic booms, while Book and Bradley (1990) observed panic and aggression in turkeys exposed to noise.

### 4.2 Effect of noise on haematology and physiology

**Figure 3:**
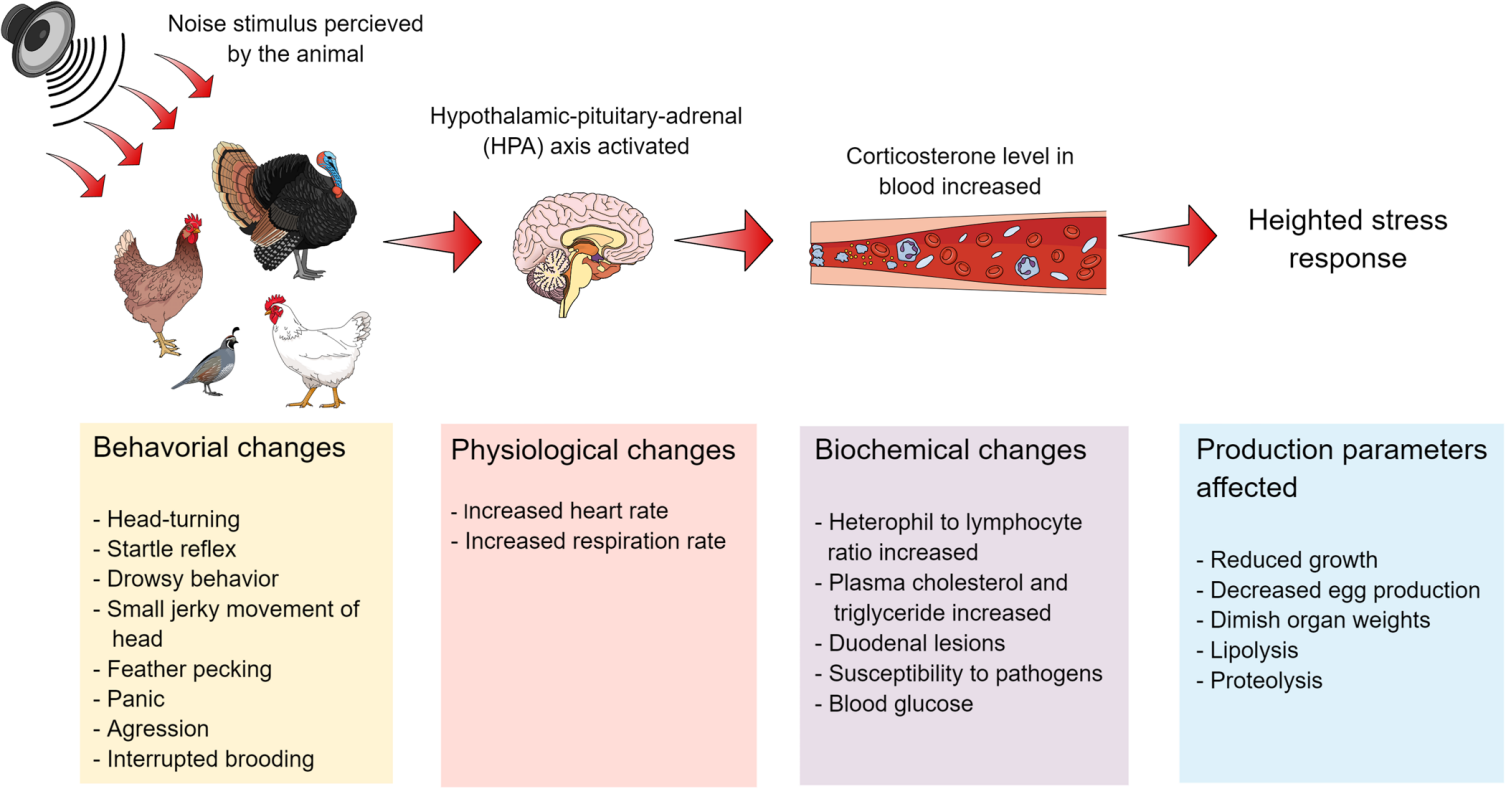
Summary of the potential effects of noise stress on poultry. The current body of literature indicates that birds exposed to noise stressors mostly exhibit behavioural modifications. Moreover, physiological responses, including a decrease in production parameters, have also been associated with birds following noise exposure. [Color figure can be viewed at wileyonlinelibrary.com]

The impact of noise on the haematology of poultry is significant and detrimental, although research in this area is limited. Extended exposure to intense noise has been linked to heightened activity of the autonomic nervous system. This sustained activation is associated with increased activity in the hypothalamic‐pituitary‐adrenal axis, elevated metabolic rates, higher blood pressure, and tachycardia (Ames, 1978; Morgan & Tromborg, 2007). It is well known that the HPA axis is a pivotal component of the endocrine system, instrumental in maintaining homoeostasis. It comprises three primary components: the hypothalamus, which synthesizes hormones such as dopamine and CRH; the pituitary gland, specifically its anterior lobe, which secretes ACTH; and the adrenal gland. The cascade release of hormones are headed by the activation of the hypopthalamus which secretes CRH followed by stimulation of the pituitary gland to release ACTH (National Research Council (US) Committee on Recognition and Alleviation of Distress in Laboratory Animals, 2008). The adrenal gland is further subdivided into the adrenal medulla, responsible for the release of catecholamines such as epinephrine and norepinephrine, and the adrenal cortex, which produces steroid hormones including cortisol, corticosterone, and aldosterone (Hall & Hall, 2020). Adrenal cortex is stimulated by ACTH to secrete the glucocorticoids. Corticosterone is the primary stress hormone in birds (Cockrem, 2007). The production of such hormones in the body is increased manifolds during stress response (Kight & Swaddle, 2011). An acute surge in these hormone levels induces metabolic changes within cells, initiating processes such as lipolysis and proteolysis, which can potentially elevate blood glucose levels (Jarosiewicz & Słowiński, 2011). During extended periods of stress, elevated blood levels of glucocorticoids contribute to disorders that diminish organ weight (e.g., lymph nodes, spleen), thereby compromising the animal's immune function and increasing susceptibility to pathogens (Frindt et al., 2006). The pathophysiology of noise stress in brief is depicted in Figure 3.

In a study by Borg (1981), it was observed that noise exposure resulted in a gradual increase in baseline steroid levels in White Leghorn hens. However, McFarlane, Curtis, Simon et al. (1989) found that continuous exposure to noise at 80 or 90 dB in broilers did not significantly affect heterophil lymphocyte ratio or plasma corticosterone levels. Nevertheless, the same authors also reported that the percentage of monocytes and the severity of duodenal lesions caused by coccidiosis increased in birds exposed to continuous noise at 80 or 95 dB. Furthermore, a significant elevation of basophil granulocytes was seen when broilers were treated with 70 and 80 dB sound intensities (Bedanova et al., 2010). Gross (1990) noted that exposure to a sound of 104 dB for 30 s increased the heterophil‐to‐lymphocyte ratio in chickens. Campo et al. (2005) also reported that laying hens exposed to noise had a significantly higher heterophil lymphocyte ratio. Chloupek et al. (2009) found that broilers exposed to noise intensities of 80 dB and 100 dB had significantly elevated plasma corticosterone levels, although exposure to these noise stimuli did not affect glucose levels and triglyceride concentrations. However, exposure to noise stimuli of 100 dB intensity resulted in a significant increase in cholesterol and triglyceride levels. Furthermore, poultry exhibited increased heart and respiratory rates, coupled with heightened secretion of stress hormones, in response to noise during transportation (Oh et al., 2011).

## 5 VIBRATION STRESS IN POULTRY PRODUCTION

Any repetitive motion occurring at regular intervals is termed vibration or oscillation (Rao, 2008). Mechanical vibrations are an inherent aspect of poultry farming, originating from the hatchery and persisting throughout all stages of production. The major role of vibration stress in the behaviour and physiology of animals comes into play during transport (Gebresenbet & Eriksson, 1998; Warriss, 1996). Motion sickness and discomfort are well‐acknowledged adverse outcomes precipitated by the movement of vehicles (BSI Group, 1987). During transport, particularly over rugged terrain, animals experience significant transmission of vehicle floor vibrations. This displacement of their centre of gravity disrupts their body equilibrium, resulting in discomfort, compromised welfare, and ultimately, a decline in meat quality (Randall, 1992). Various intrinsic factors such as body weight, size, age, gender, fitness level, and body posture govern an individual's response to vibration stress (Griffin, 2012). Individual organisms are known to exhibit diverse responses to incoming vibrations (Oborne et al., 1981). The peak vibration levels occur at the resonance frequency, where the body absorbs maximal mechanical energy compared to any other frequency. When exposed to vibrations, body organs function as a heterogeneous ensemble of mechanical systems, resulting in the displacement of internal organs (Pape et al., 1963). Studies have shown that the fundamental frequencies of the floor in bird containers typically range between 1 and 4 Hz, with an additional peak observed at 10–12 Hz (Behrends et al., 1996; Randall et al., 1993). These frequencies closely align with those known to affect a 2 kg broiler, potentially triggering vibration stress responses (Randall et al., 1996). Furthermore, the siting of animal farms near workshops, railways, construction sites, and other such areas can subject them to stress induced by ground‐borne vibrations (Neil & McKay, 2003). Additionally, an array of sources contributes to vibrations, such as ventilation hoods, refrigerators, elevators, exhaust fans, and a variety of other motor‐driven equipment. These vibrations significantly disrupt the poultry production chain. While little is known about vibration stress physiology, it is closely related to noise stress, as they are both energy waves (Reynolds et al., 2018). It impacts the quality of broiler meat during transportation to slaughter (Abeyesinghe et al., 2001; Carlisle, 1998; Randall et al., 1993; Randall et al., 1997).

### 5.1 Effect of vibration on eggs

The loss of table eggs or fertile eggs due to cracks, breaks, and changes in internal quality are some noticeable effects of vibrations on poultry eggs (Berardinelli et al., 2003a, 2003b; Nazareno et al., 2013; Torma & Kovácsné, 2012). The exposure of eggs to mechanical vibrations can cause changes to the integrity of the eggshell, yolk, and albumen, leading to cracks in eggshells, liquefaction of albumen, a reduction in Haugh unit values, and decreased resistance of the yolk's membrane. These changes demonstrate the detrimental effects of vibrations on the internal quality of eggs (Berardinelli et al., 2003a, 2003b). An early landmark study focused on fertilized eggs, where exposing them to daily vibrations at a rate of 3600 movements per minute for 15 min before the incubation period showed no impact on hatchability Proudfoot (1969). However, Taggart et al. (1990) reported a reduction of up to 68% in hatching rate for eggs exposed to vibration. Additionally, the authors noted that eggs subjected to vibrations may suffer from broken yolks or altered primary structures of the fertilized disc, preventing successful development. In their seminal study, Shannon et al. (1994) explored the effects of mechanical vibrations spanning frequencies from 5 to 50 Hz and accelerations ranging from 0.09 to 4.93 m/s² on fertilized eggs. They reported a mortality rate of 31.9%, highlighting that both the amplitude and frequency of vibration exposure correlated with decreased hatching success. Torma and Kovácsné (2012) documented lower hatchability and increased incidence of embryonic abnormalities in eggs subjected to vibrations, underscoring the detrimental impact of such environmental stressors on avian embryonic development. It is important to note that eggs in the production chain are constantly exposed to mechanical impacts during various operations such as collection, packaging, and shipping (Altuntaş & Şekeroğlu, 2008). Nazareno et al. (2013) concluded that mechanical vibrations directly influence pre‐farm losses. The high vibration levels on both asphalt and land roads can result in cracked and broken eggs. Mechanical vibrations deteriorated the hatchability and hence reduced the number of high‐quality day‐old chicks obtained (Donofre et al., 2017).

### 5.2 Effect of vibration in broilers

Transportation of broilers over long distances can induce significant stress, exacerbated by the distance between farms and slaughterhouses (Warriss et al., 1997). During transport, birds endure a spectrum of physical and psychological stressors, resulting in compromised welfare and production performance (Mitchell & Kettlewell, 1998; Schwartzkopf‐Genswein et al., 2012; Warriss et al., 1997). Randall et al. (1993) and Carlisle (1998) reported that vibrations caused fear and discomfort in birds, leading to negative effects on meat quality. In practical conditions, Randall et al. (1993) found that vibrations with frequencies up to 10 Hz on the vertical axis and 18 Hz on the lateral axis occurred during the transportation of chickens by trucks for slaughter. Since internal organs are not firmly anchored within the body cavity, they can experience relative movement when subjected to vibrations. This movement is relative to the mass of the internal organs (Scott, 1994). When induced to resonate, organs may suffer damage varying from minor cell rupture to severe impairment of function and internal bleeding. Thus, the resonance of internal organs could pose a notable aversive stimulus to birds during vibration exposure. Road transportation has been demonstrated to trigger stress responses in broilers, evidenced by an increased heterophil‐to‐lymphocyte ratio and elevated plasma kinase levels (Mitchell et al., 1992). It was also reported that vibrations between 2 and 10 Hz caused a significant reduction in muscle glycogen in birds (Warriss et al., 1997). Additionally, Carlisle (1998) found that different vibration levels (2, 5, 10 Hz) resulted in higher cortisol levels in birds. Studies indicate that vibration stress induces biochemical changes, leading to elevated concentrations of brain neurotransmitters (Ariizumi et al., 1986; Kosmakos et al., 1975). However, Garcia et al. (2009) reported no change in rectal temperature and weight loss in broilers exposed to simulated transportation vibrations. Abeyesinghe et al. (2001) suggested that birds tend to avoid vibrations because they cause fear among the birds. As the vibration is perceived as a stress stimulus to the birds, it tends to evoke stress responses in the body. This could be a possible explanation for higher cortisol levels in birds when subjected to different levels of 2, 5, and 10 Hz (Carlisle, 1998).

Studies have also observed that newly hatched chicks naturally prefer consonant sounds over dissonant ones (Chiandetti & Vallortigara, 2011). In biological terms, the function of fear is to guide birds away from potential harm, thus serving as an advantageous survival mechanism. However, under certain circumstances, fear can become detrimental if birds are unable to escape from the distressing stimulus. (Jones, 1996; Mills & Faure, 1990). This fearfulness is influenced by a variety of genetic and epigenetic factors. The impact of environmental influences, particularly in the early developmental stages, is paramount (Boissy, 1995). The degree of fearfulness is significantly shaped by external stimuli encountered during early life stages. Hence, raising poultry in a complex environment can reduce the degree of fearfulness (Johnsen et al., 1998).

It is impractical to believe that environmental noise can be completely eliminated in poultry rearing conditions. Additionally, controlling the quality of roads and trucks during transport is not always feasible. Consequently, it is necessary to develop and implement strategies based on new scientific research to mitigate vibrations in all poultry farm operations, from prehatch to farm and farm to fork, by utilizing engineering concepts to design new vehicle boxes, trays, and materials. To minimize stress caused due to noise and vibration, it is essential to strategically position and operate ventilation fans and feeding equipment with precision. Moreover, conditioning of birds may help to decrease their fearfulness. Systematic desensitization, which has been successfully used to treat phobias in humans (Lang & Lazovik, 1963), should also be validated in birds for both natural and pathological phobias. Some chicken strains are particularly sensitive to noise and may react with panic to even minor sounds, resulting in significant losses (Korsós et al., 2019). Hence, sensitivity and display of fearful behaviour could be controlled by various genetics and epigenetics factors. It is essential to study such relations before deducing a way to mitigate vibration stress. Therefore, a holistic approach that considers the bird's tolerance level, as well as the different stages of production and transport, may provide effective solutions for mitigating the adverse effects of noise and vibration in poultry production.

## 6 LIGHTING AND PHOTOPERIOD STRESSORS

### 6.1 Disruptions in natural lighting

Over the years, it has been generally believed that variations in light ratio have a greater influence on egg production in poultry than quantitative factors derived from the actual amount of light present. As light plays a critical role in triggering the secretion patterns of various hormones, it significantly impacts growth, maturation, and reproduction in poultry. Inadequate light management can result in visual anomalies, skeletal deformities, and circulatory problems, among other deficiencies (Manser, 1996; Olanrewaju et al., 2006). To improve poultry production and efficiency, it is essential to address aspects of environmental management, such as light intensity, duration, wavelength, and the photoperiodic regime, as these factors significantly affect the physical activity of chickens (Lewis & Morris, 1998; Olanrewaju et al., 2006).

**Figure 4:**
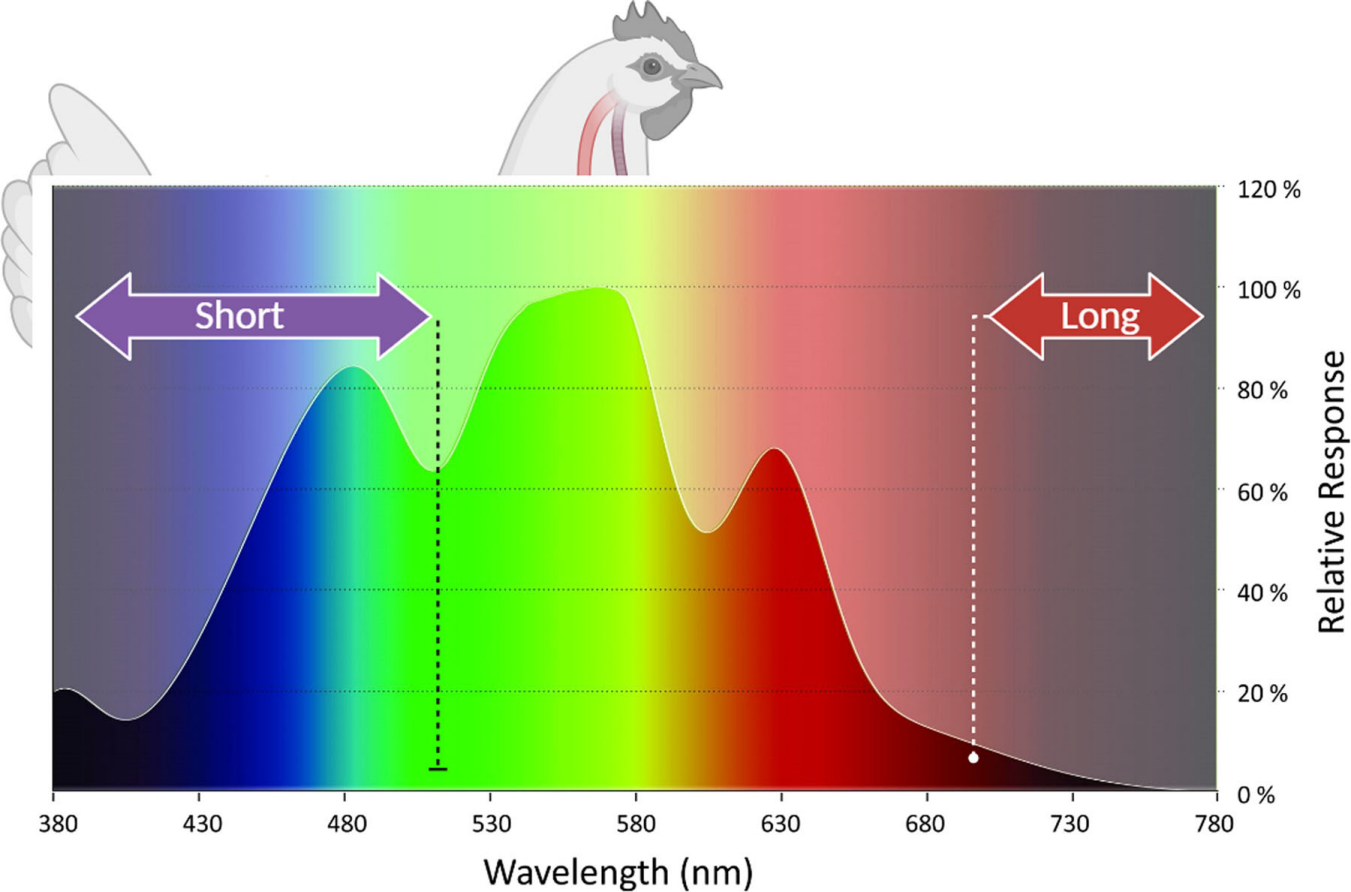
Relative response of poultry species to photopic conditions in terms of spectrum. Adapted from a previous study (El‐Sabrout et al., 2022) and from ONCE (Stephan, 1985 retrieved at www.once.lighting/en/news/top-3-reasons-why-we-prefer-chicken-luxover-lux). Photopic variations were executed using different light‐emitting diodes from short to long wavelengths. [Color figure can be viewed at wileyonlinelibrary.com]

Recent findings indicate that the wavelength of light (El‐Sabrout et al., 2022) affects the ability of poultry to respond to stress, fear, and depression during their comfort phase (Figure 4). This light exposure can affect hatching rates when embryos are exposed to sunlight during incubation (El‐Sabrout, 2017; Shafey, 2004; Yu & Li, 2023). Specifically, exposure to green, red, and white light spectra can reduce fear and sensitivity in broilers (Archer, 2017). Irregular timing of light exposure can cause circadian disruption by affecting the suprachiasmatic nuclei (SCN) of the hypothalamus; subsequently impacting the coordination of physiological functions and behaviours in humans and animals (Nicholls et al., 2019). Additionally, daily rhythm‐associated group‐specific dynamics, such as flocking in birds and synchronized feeding in domestic fowl, can improve oscillatory brain activity in the alpha and mu bands, enhancing brain‐to‐brain synchronization at the dyadic level (Collins & Sumpter, 2007; Gordon et al., 2020). Soliman and El‐Sabrout (2020) provided their conclusions by citing studies that highlighted significant anatomical regions influencing the visual function of poultry. (1) The retina helps achieve proper growth and behaviour by responding to environmental light (Lewis, 2006; Wilson & Lindstrom, 2011); (2) the pineal gland, responsible for secreting melatonin and serotonin hormones that regulate mobility, body temperature, and mating seasons (Baxter et al., 2014; Lewis, 2006) and; (3) the hypothalamus, which directly controls the gonadotropin‐releasing hormone (GnRH), homoeostasis, and physiological structures based on light intensity, with short wavelengths necessary at high densities (Baxter et al., 2014). Therefore, light colour from different wavelengths, including the invisible spectrum, affects the well‐being of poultry, influencing behavioural responses, particularly fear behaviour (Biyatmoko, 2014; Riber, 2015). It is also necessary to consider other impacts, as shown in Table 1.

**Table 1:** Impact of various light colours and wavelengths on poultry behaviour, health, and production.

Light colour and wavelength (nm)	Poultry Species	Results	References
Yellow (600 nm) and white (400–700 nm)	Cherry Valley ducks	Spent more time walking, ground pecking, drinking, and social interactions	Sultana et al. (2013)
Blue (460 nm) and green (520 nm)		Spent more time sitting, and standing, and reduced fear response	
Red (625 nm)	Brown Tsaiya ducks	Higher egg performance due to higher estradiol concentrations	Su et al. (2021)
Red (618–635 nm)	Japanese Quails	Higher percentages of fertility (90.07%) and total egg hatchability (83.47%)	Elkomy et al. (2019)
Red (660 nm)	Cobb broiler breeders	Stimulate the production of gonadotropin‐releasing hormone‐I (GnRH‐I) that led to greater egg production	England and Ruhnke (2020); Mobarkey et al. (2010)
Combination of red and (618–635 nm), green (515–535 nm)	Hy‐Line Brown laying hens	Increased levels of reproductive hormones, ovarian weight, and follicle numbers	Hassan et al. (2013)
Green light (560 nm) and blue light (480 nm)	Arbor Acres male broilers	Improved cellular and humoral immune responses and alleviated stress response in broilers due to decreased in Interleukin‐1 beta (IL‐1*β)* serum levels	Xie et al. (2008)
Green light (560 nm)		Obtained heavier muscle weight due to higher levels of circulating insulin‐like growth factor‐I (IGF‐I)	Liu et al. (2010)

### 6.2 Light intensity or illuminance

The light intensity in poultry houses and other artificial environments is measured in Lux or cd m^‐2^ units and has a significant impact on the behaviour of chickens. As per the standard broiler lighting regimen, a continuous light intensity of 20 lux is maintained during the initial 7 days (early posthatch period), after which it is adjusted to a dim intensity of 3–5 lux for the remaining rearing days (National Chicken Council, 2010).

Artificial light sources typically generate light with an alternating current of 50 Hz/s, resulting in fluctuations in light intensity as lamps turn on and off 100 times per second (Ksenzhek & Volkov, 1998). The use of brighter light (>5 lux) can lead to increased chicken activity and decreased body weight (Buyse et al., 1996). Increased activity can potentially reduce the occurrence of skeletal and metabolic disorders (Charles et al., 1992). Broiler chickens exposed to intensified natural light (85.2 lux) exhibited increased activity, as evident from elevated frequencies in standing, locomotion, eating, and ground pecking throughout the 2–6 weeks of observation. This observation implies that avian vision evolved under natural light conditions, enabling them to perceive flickering light of higher frequencies (Rubene, 2009). This ability is attributed to their potential tetrachromatic vision, theoretically allowing them to distinguish twice as many colours as humans (Goldsmith, 1990). Therefore, the flicker sensitivity of domestic poultry birds could easily discourage them from approaching specific objects that might induce discomfort and stress, particularly within the frequency range of 39–71 Hz, and under luminance levels between 10–1000 cd m^−2^ (Jarvis et al., 2002). The growth rate of Ross broilers reared at 20 lux, which promoted higher behavioural activity, was lower than that of broilers raised at 5 lux, resulting in reduced eye weight and weight gain. (Rault et al., 2017). However, gradually reducing light intensity from 25 lux for the first 7 days to 2–5 lux can serve as a preventative measure to enhance broiler performance in terms of cannibalism and feather pecking, as well as reduce walking and standing behaviours (Buyse et al., 1996). Other researchers have also suggested that fluctuations in natural light intensity and quality throughout the day can synchronize behaviour within flocks by affecting circadian and ultradian rhythms, potentially influencing leg health. For instance, broilers housed in 200 lux spent more time preening, and promoting healthy feathers, during the entire photoperiod than those housed in 5 lux. However, this difference in light intensity did not affect leg health (Alvino et al., 2009; Kristensen et al., 2006).

In laying hens, it was found that exposure to 5 and 100 lx from LED light did not have an impact on the physiological production performance or egg quality of the Hy‐Line W‐80 strain. However, it was observed that illumination exposure starting from Days 7 to 28 influenced cloacal and rectal temperature, feed intake, body weight, and albumen percentage. As a result, it was concluded that 5 lux and 28 days of acclimation is ideal for laying hens (Bahuti et al., 2023). Another study observed that light intensity of 99 up to 323 lux, with an 83.8 cm distance between bulbs and feeders, in Lohmann‐Brown hens stimulates earlier sexual maturity, resulting in higher egg production but decreased egg weight (59.6 g/bird) (Erensoy et al., 2021). Additionally, the specific gravity of eggs in Hy‐Line W‐36 laying hens is directly related to egg quality. This quality can be enhanced by exposure to 28 lux LED light at night, which has been shown to improve productive performance (Ribeiro et al., 2021). Recent research has shed light on the connections between light intensity and wavelength, as summarized in Table 1. Additionally, the impact of illuminance, measured in lux, on the growth and behaviour of broilers was also evaluated (Table 2).

**Table 2:** The impact of varying light intensities measured in lux on broiler performance and behaviour.

Flock age (weeks)	Light Intensity (lux)	Result	References
2–6	5	Live weight ↑; Feed conversion ratio ↓	Ahmad et al. (2011)
0–8	40	Dustbathing behaviour ↑; Walking behaviour ↑	Kang et al. (2023)
1–5	5	Triglyceride levels in the serum ↑; creatinine ↓; Interleukin‐6 content ↑; Corneal diameter ↑; Physiological stress ↑	Kim et al. (2022)
0–6	5	Feed efficiency ↓	Aldridge et al. (2022)
	10	Feeding behaviour ↑	
	20		
0–6	1	Activity ↓; Body weight ↑; Eye size ↑; Activity during scotophase ↑	Blatchford et al. (2012)
	50 or 200	Activity during photophase↑	
0–4	1	Eye weight and size ↑; ulcerative footpad lesions↑	Deep et al. (2010)
0–2	≥5	Metabolism ↑; Growth ↑; Welfare ↑	Arowolo et al. (2019)
1–6	<1	Corneal diameter of eye ↑; Body weight ↓	Yang et al. (2018)
	>10	Mortality ↑; Uniformity ↑; response to T3 hormone	
	30–200	Feed intake ↑	
0–6	10	Blood glucose level ↓; Size of bursa of Fabricius ↑	Bayraktar et al. (2012)
	5		
5–6	20	Feed and water consumption ↑	Raccoursier et al. (2019)
	5	Densities away from feed ↑	

*Note*: “↑” refers to an increase in a parameter measured; “↓” refer to a decrease in a parameter measured.

### 6.3 Photoperiod regime or lighting programmes

Beyond the conventional factors such as temperature, humidity, air velocity, and radiation, the environmental photoperiod, particularly the 24‐h Light‐Dark (LD) cycle, provides crucial information about daily sunlight changes necessary for maintaining and synchronizing physiological and behavioural homoeostasis (Jiang et al., 2023). Notably, a long photoperiod (20 L:4D) in Ross 308 broiler chickens has been reported to deteriorate meat quality, whereas a near‐constant photoperiod (23 L:1D) elevates stress and fear levels (Wang et al., 2008). Furthermore, near‐constant photoperiods disrupt circadian clock gene expression and cecal microbiome diversity in Hy‐Line Brown Layer chickens, with early‐life photoperiod‐driven circadian rhythms being linked to gut health (Hieke et al., 2019). Given these findings, establishing a well‐planned light schedule is imperative to maximize poultry growth and well‐being, ultimately leading to economic benefits (Jiang et al., 2023; Wu et al., 2022). The length of time that hens spend in the light—also called their lighting regimen—has a big impact on the kind of meat or eggs that they lay (Olanrewaju et al., 2006). Additionally, it can increase pullet flock egg output, which is advantageous for both commercial and breeding layers (Reddy et al., 2014). Various lighting programmes substantially impact performance, immunity, and the secretion of hormones that control growth and reproduction (Zheng et al., 2013). Consequently, photoperiod regulation is manipulated to enhance growth and economic performance while ensuring bird health (Moraes et al., 2008). This phenomenon has been substantiated through experiments assessing the locomotor activity of broilers under quasicontinuous light (23 L:1D) and 16 L:8D conditions. The average activity level during the light phase increased as the duration of light decreased. Broilers exposed to 23 L:1D failed to establish a circadian activity pattern, unlike those under 8D:16 L, highlighting the importance of circadian rhythms as an indicator of welfare in domestic animals (Bessei, 2006; Reiter & Bessei, n.d.).

Research has demonstrated that exposure to bright light (40 lux; 17D) mitigates issues related to leg and eye health, positively impacting cold carcasses, whole breast meat, and wing yields (Deep et al., 2010). Conversely, lighting programmes incorporating extended darkness (approximately 7 h per day) improve broiler welfare by reducing the number of birds experiencing pain (Schwean‐Lardner et al., 2013). In broilers, light exposure is essential for encouraging skeletal development through feed intake, which has a substantial effect on overall performance. This involves affecting the ability of chicken embryos to respond to predators, namely tonic immobility (Archer & Mench, 2017). When evaluating fearfulness in birds, particularly poultry, TI is a useful metric (Gallup, 1974). A study demonstrated that chickens exposed to 23 h of light (23 L) exhibited a longer duration of TI on Day 10, although the trend reversed on Day 36. Birds exposed to increasing light (24 L for the first 3 days) had a longer duration of TI at that point. This suggests that birds exposed to interrupted lighting (IL) may face challenges in adapting to changes in day length in later life, resulting in heightened fear responses to humans (Wang et al., 2008). Continuous light exposure (24 L) negatively impacted embryonic leg development, diminishing leg bone strength later in life (van der Pol et al., 2019). Conversely, administering a daily cycle of 12 L:12D during embryogenesis led to a sustained decrease in fearfulness and positively influenced long‐term leg health (Archer & Mench, 2017). These effects have significant economic implications, affecting factors such as marketing time.

## 7 CONCLUSION

Although the current literature remains unbalanced, it has contributed significantly to the understanding of the general physiology of stress. Abiotic stressors can lead to changes in metabolism, behaviour, and immunity. Chronic stress can result in neurobiological alterations that impact brain structure. While pathogens can be managed with common prophylactic measures, abiotic factors such as temperature and noise can be more challenging to control. Climate change is expected to exacerbate these issues, with more extreme temperature episodes and noise disruptions expected in the future. Additionally, disruptions in lighting regimens or inadequate light intensity can negatively impact layer production performance. While research on temperature‐related stressors has improved, a nuanced understanding of all abiotic stressors is necessary for sustainable poultry production systems. The scientific community must also explore the complex interactions between poultry and their environments to develop effective solutions.

## AUTHOR CONTRIBUTIONS

All authors contributed substantially to the preparation, writing, and revision of the manuscript.

## CONFLICT OF INTEREST STATEMENT

The authors declare no conflict of interest.

## Data Availability

The current manuscript is a review article hence, it is not applicable.
